# A Molecular CO_2_ Reduction Catalyst Based on Giant Polyoxometalate {Mo_368_}

**DOI:** 10.3389/fchem.2018.00514

**Published:** 2018-11-02

**Authors:** Santu Das, Tuniki Balaraju, Soumitra Barman, S. S. Sreejith, Ramudu Pochamoni, Soumyajit Roy

**Affiliations:** ^1^Eco-Friendly Applied Materials Laboratory, College of Chemistry, Central China Normal University, Wuhan, China; ^2^Eco-Friendly Applied Materials Laboratory, Department of Chemical Sciences, Materials Science Centre, Mohanpur, Indian Institute of Science Education & Research, Kolkata, India

**Keywords:** CO_2_ reduction, polyoxometalate, homogeneous catalysis, water oxidation, photochemistry

## Abstract

Photocatalytic CO_2_ reduction in water is one of the most attractive research pursuits of our time. In this article we report a giant polyoxometalate {Mo_368_} based homogeneous catalytic system, which efficiently reduces CO_2_ to formic acid with a maximum turnover number (TON) of 27,666, turnover frequency (TOF) of 4,611 h^−1^ and external quantum efficiency of the reaction is 0.6%. The catalytic system oxidizes water and releases electrons, and these electrons are further utilized for the reduction of CO_2_ to formic acid. A maximum of 8.3 mmol of formic acid was observed with the loading of 0.3 μmol of the catalyst. Our catalyst material is also stable throughout the reaction. The starting materials for this experiment are CO_2_ and H_2_O and the end products are HCOOH and O_2_. The formic acid formed in this reaction is an important H_2_ gas carrier and thus significant in renewable energy research.

## Introduction

The CO_2_ concentration in environment is ever increasing. Thus, to find out a suitable pathway to recycle CO_2_ to an energy rich material is a crucial challenge nowadays (Hoffert et al., 2002; Crabtree and Lewis, 2007; Meinshausen et al., 2009; Mikkelsen et al., 2010; Garai et al., 2012; Bandeira et al., 2015; Twidell and Weir, 2015). The depletion of fossil fuel during production of energy increases the CO_2_ level in the environment (Hoel and Kverndokk, 1996; Höök and Tang, 2013). It is also known that abundance of fossil fuel is limited. Thus, it is necessary to find out a new pathway which can produce energy without hampering the environment and burning fossil fuels. With this end in view if we convert a greenhouse gas like CO_2_ to energy rich material it would be very interesting and helpful for a sustainable environment and economy (Khenkin et al., 2010; Rankin and Cummins, 2010; Wang W. et al., 2011; Bontemps et al., 2012; Kuhl et al., 2012; Ohtsu and Tanaka, 2012; Wesselbaum et al., 2012; Xi et al., 2012; Arai et al., 2013; Costentin et al., 2013; Asadi et al., 2014; Blondiaux et al., 2014; Herrero et al., 2014; Kim et al., 2014; Kou et al., 2014; Lu et al., 2014; Studt et al., 2014; Zhang et al., 2014; Gao et al., 2015; Kornienko et al., 2015; Liu et al., 2015; Marszewski et al., 2015; Matlachowski and Schwalbe, 2015; Roberts et al., 2015; Sypaseuth et al., 2015; Iwase et al., 2016; Kuriki et al., 2017). However, the challenge is as CO_2_ is a very stable oxide of carbon at its stable oxidation state, a large amount of energy is required to activate CO_2_. Activation of CO_2_ is a challenge. Here this challenge is addressed via photochemical reduction of CO_2_ to HCOOH using an unusually large giant POM cluster, first synthesized by Müller group in Bielefeld {Mo_368_} (Müller et al., 2002, 2004; Müller and Roy, 2003). The TON (27,666) and TOF (4,611 h^−1^) of this conversion is quite high as compared to other reported molecular catalysts (Tamaki et al., 2015). In photosynthesis, nature continuously activates CO_2_ with ease where CO_2_ from environment is converted into carbohydrates through photosensitization using sun as the source of light. The process happens in nature such that first water gets oxidized and releases electrons which further reduce CO_2_ to carbohydrate in a long catalytic cycle (Hatch, 1976). Drawing inspiration from this process, use of a proper catalytic system can lead to the conversion of CO_2_ to different high energy carbon material under light. Many potential catalytic systems have been developed over the last few decades for the synthesis of different fuel and organic materials from CO_2_. Some of the catalysts initially bind with CO_2_ and further reduce it to different reduced materials (Castro-Rodriguez and Meyer, 2005; Laitar et al., 2005; Sakakura et al., 2007; Sadique et al., 2008; Cokoja et al., 2011; Langer et al., 2011; Mandal and Roesky, 2011; Sato et al., 2011; Schmeier et al., 2011). Some catalysts convert CO_2_ following electrochemical methods (Amatore and Saveant, 1981; Hori et al., 1989; Whipple and Kenis, 2010; Agarwal et al., 2011; Finn et al., 2012; Kuhl et al., 2012; Sullivan et al., 2012; Costentin et al., 2013; Zhang et al., 2014; Kornienko et al., 2015; Lin et al., 2015). Electrochemically CO_2_ can be reduced to different alkane, ethylene, CO, and HCOOH. The major problem associated with this process is the selectivity in the reduction of CO_2_ to different reduced products. Recently Yaghi group showed that electrochemically CO_2_ can be reduced to CO selectively using Co-porphyrin based COF and MOF catalysts in water (Kornienko et al., 2015; Lin et al., 2015). Imminent challenge lies in the photochemical reduction of CO_2_ (Matsuoka et al., 1993; Daniel and Astruc, 2004; Schwartzberg and Zhang, 2008; Takeda et al., 2008; Li and Zhang, 2009; Morris et al., 2009; Dhakshinamoorthy et al., 2012; Tornow et al., 2012; Sato et al., 2013; Sekizawa et al., 2013; Zhu et al., 2013; Wang et al., 2014; Kim et al., 2015; Li et al., 2015; Low et al., 2015). Among various proposed technologies, photocatalytic CO_2_ reduction has been known as one of the most important strategies for solving both global energy and environmental problems due to its low cost, cleanliness, and environmental friendliness (Maginn, 2010; Iizuka et al., 2011; Sato et al., 2011; Yu et al., 2014). Lehn (Hawecker et al., 1984) and Sauvage (Beley et al., 1984) started photochemical and photoelectrochemical CO_2_ reduction in 1980 using different rhenium, ruthenium, nickel, and cobalt complexes of different macrocycles as catalysts (Fisher and Eisenberg, 1980; Beley et al., 1984). Many hybrid nano materials are potential catalysts for this purpose. Also, some ruthenium and rhenium-based metal complexes can reduce CO_2_ in presence of light. Photo-electrochemical method is another important tool in this regard (Halmann, 1978; Barton et al., 2008). The major problem associated with photochemical CO_2_ reduction is the use of a sacrificial electron donor, an organic amine, which cannot be recovered from the reaction (Takeda et al., 2008; Morris et al., 2009). One of the interesting solution of this problem is using water as a sacrificial electron donor (Wang C. et al., 2011; Kim et al., 2014). However, all such avenues for CO_2_ reduction suffers from low yield of the reduced product. Moreover, the catalyst materials are also expensive. Thus, it is necessary to develop a catalyst which is inexpensive, easy to synthesize and can reduce CO_2_ in water with promising yield. Till date the photo catalyst materials used for CO_2_ reduction in water are majorly heterogeneous (Barton et al., 2008; Xi et al., 2012; Kuriki et al., 2016). Homogeneous photochemical CO_2_ reduction in water is also reported (Nakada et al., 2016).

A wide variety of catalysts both homogenous and heterogenous have been reported for the reduction of CO_2_ to formic acid ranging from macrocycles (Chen et al., 2015; Ikeyama and Amao, 2018), hybrid materials (Yadav et al., 2012; Sekizawa et al., 2013; Yoshitomi et al., 2015), ionic liquids (Lu et al., 2017), nanoparticles (Kortlever et al., 2015), B doped nanodiamonds (Ikemiya et al., 2018) to alloys (Bai et al., 2017) with some of them reaching selectivity as high as 100%. On the other hand, reports on photochemical CO_2_ reduction to HCOOH using molecular catalysts mainly employs Ru based complexes as the active catalyst (Boston et al., 2013). Use of bipyridine based Ru(II) complexes with an external light sensitizer have been reported for the selective reduction of CO_2_ to formic acid (80%) with a TON = 526 (Rosas-Hernández et al., 2015). By employing Ru based supramolecular photocatalysts which acts as both light sensitizer and catalyst, Ishitani *et al*. have showed selective reduction of CO_2_ to formic acid in the presence of an external reductant (Tamaki et al., 2012). Further they have tuned the catalytic activity of Ru(II)-Ru(II) supramolecular photocatalyst [Ru_2_-Ru(CO)] by employing a suitable reductant to increase formic acid selectivity (87%) and TON_HCOOH_ = 2,766 (Tamaki et al., 2015). Here a synergistic interaction between the reductant employed and the photocatalyst is dictating the outcome of the reaction. These catalysts have an advantage of visible light absorption but require a need for an external electron donor, which was either added externally or added as a part of the solvent. Also, a similar RuReCl photocatalyst was investigated in aqueous solution but the efficiency of formic acid production was low due to the degradation of photocatalyst owing to the back-electron transfer from one electron reduced species to the photosensitizer unit (Nakada et al., 2015). In contrast, in this work a giant {Mo_368_} POM based homogenous photocatalyst system is used to achieve higher selectivity and TON toward formic acid production by employing water solvent as the electron donor.

Recently our group has reported molybdenum based heterogeneous Soft-Oxometalate materials as efficient catalysts for CO_2_ reduction reaction (Das et al., 2016). To achieve the same in homogenous realm, an oxo-molybdate based catalyst material which is completely soluble in water is chosen. In this work a molecular catalyst based on giant molybdenum polyoxometalate {Mo_368_} is synthesized, which can reduce CO_2_ to formic acid in water with a good yield. The mixed valent molybdenum based giant polyoxometalate {Mo_368_}is synthesized following the literature procedure (Müller et al., 2002). Due to the presence of Mo^V^ and Mo^VI^ centers in the cluster, intra-valance charge transfer bands are observed in the polyoxometalate. This band imparts deep blue color to the solution. The cluster, a member of molybdenum blue family, is also photoactive and thus there is no need of an addition of any photosensitizer (Das et al., 2016a). Photoactivity of polyoxometalates is known and has been used by us as a catalyst in photo-polymerization (Chen et al., 2013; Das and Roy, 2015, 2016), as well as in photochemical water oxidation reaction (Roy et al., 2007a,b, 2008; Das et al., 2016b; Barman et al., 2018) without addition of any sensitizer and other reactions (Roy et al., 2013). The cluster {Mo_368_} is extremely efficient as a catalyst and 8.3 mmol of formic acid is obtained with a loading of 0.3 μmol of catalyst. Here the catalyst acts with a maximum turnover number (TON) of 27,666 and turnover frequency (TOF) of 4,611 h^−1^.

## Materials and methods

All the materials and reagents were purchased from commercially available source and used without further purification. Only water is used as a solvent which was distilled twice before starting any reaction. Before use, all the glass apparatus were first boiled in acid bath then cleaned first with tap water then with double distilled water and finally rinsed with acetone and dried in hot air oven overnight, the temperature of which was set at 90°C. A Luzchem UV photoreactor operating at a power of 64 W (8 × 8 W) with UVA lamp is used for photochemical CO_2_ reduction reaction in water.

### Synthesis of Na_48_[H_X_Mo_368_O_1032_(H_2_O)_240_(So_4_)_48_]. ca. 1,000H_2_O

{Mo_368_} is synthesized by following literature procedure. To a solution of Na_2_MoO_4_·2H_2_O (3 g, 12.4 mmol) in water Na_2_S_2_O_4_ (0.15 g, 0.86 mmol) is added as a reducing agent. The reaction mixture is acidified with 0.5 M H_2_SO_4_ (35 mL; immediate color change to blue). The solution is then stored in a closed flask for 2 weeks and after 2 weeks the precipitated deep-blue crystals of {Mo_368_} are obtained by filtration, Yield: 135 mg.

### General reaction procedure for photo catalytic CO_2_ reduction

Photo catalytic carbon dioxide reduction reactions are carried out as follows. Desired amount of {Mo_368_} is taken in 10 ml of oxygen free double distilled deionized water. The reaction mixture is closed in a two neck round bottom flask and CO_2_ gas is purged for 2.5 h. Then the reaction mixture is kept in the photo reactor under UV-light (eight 8 W lamps, λ = 373 nm) for different intervals of time. 20 μL of reaction mixture is taken out and further diluted with 10 ml HPLC grade water, which is used for carrying out MALDI-MS experiment. For MALDI-MS experiments the reaction mixture is co-crystallized with HCCA matrix and then the mass spectrum was recorded. Mass spectrum gives a molecular ion peak of formic acid (m/z = 46; Figure S1). Further to detect formic acid, HPLC measurement was performed by injecting the diluted reaction mixture in carbohydrate column with an external standard, 0.1 M formic acid solution. and all the quantitative measurements of formic acid is done by using HPLC experiments. CV measurements with the reaction mixture are done using 0.1 M KCl as an electrolyte, in a potential range of +0 to −2 V with respect to saturated Ag/AgCl reference electrode in a standard 3-electrode system and a peak around −0.59 V in cyclic voltammogram further confirms the formation of formic acid from carbon dioxide in our reaction mixture. To further prove formic acid is formed in our reaction mixture, a coupling reaction is performed with our reaction solution using the following method. A solution of aniline (100 μL) in 2 ml acetonitrile along with 20 mg HATU is added to the reaction mixture as a coupling reagent. The reaction mixture is stirred for 2 h at room temperature. The N-phenylformamide is formed in the reaction mixture. Organic components are extracted with EtOAc (3 × 15 ml) and the EtOAc is evaporated in vacuum. The extracted organic component is dissolved in acetonitrile to perform GC-MS and MALDIMS analysis by following above mentioned procedures. Also we have taken the proton NMR spectrum of amide in CDCl_3_ solution to further confirm the formation of amide (Figure S1).

### Determination of oxygen using YSI dissolve oxygen meter

Oxygen formed in the reaction is detected by YSI dissolved oxygen meter. YSI dissolved oxygen meter is first calibrated by degassed water. For that purpose, Nitrogen gas is first bubbled through double distilled water for an hour and then put YSI dissolved oxygen meter into this water and recorded the amount of oxygen present. Next, dissolved oxygen meter is dipped into the reaction system i.e., photo illuminated sample and amount of oxygen present in the reaction system is recorded. Now from the difference of oxygen reading in YSI dissolved oxygen meter the total oxygen formed in the reaction is calculated.

#### pH dependent reaction

These experiments are performed by following the previous procedure using different buffer solutions in the range of pH 5 to 9. Measurement of the formic acid is carried out by similar methods as mentioned above.

### Characterization techniques

#### Fourier transform infra-red spectroscopy (FT-IR)

FTIR spectrum of {Mo_368_} is performed by KBr pallet technique. Initially a pellet is prepared from the mixture of KBr and {Mo_368_}. FTIR spectrum is recorded by using Perkin Elmer Spectrum RX1 spectrophotometer with FTTR facility in the range 2,000–450 cm^−1^.

#### Electronic absorption spectroscopy (EAS)

A stable solution of {Mo_368_} is taken in a quartz cuvette and the electronic absorption spectrum is recorded on U-4100 Spectrophotometer (Liquid).

#### Cyclic voltammetry

PAR model 273 potentiostat is used for CV experiment. A platinum wire auxiliary electrode, a glassy carbon working electrode with surface area of 0.002826 cm^2^ and an aqueous Ag/Ag^+^ reference electrode which is filled with saturated KCl solution, is used in a three-electrode configuration. All the measurements were performed at 298 K in an inert atmosphere.

#### Raman spectroscopy

A LABRAM HR800 Raman spectrometer is employed using a He–Ne ion laser (λ = 1,024 nm) as the excitation source to analyse the sample.

### HPLC

All reaction samples were monitored by HITACHI- HPLC system equipped with binary 2,130 pumps, a manual sampler, and 2,490 refractive index detector, maintained at 50°C. The products were separated in sugar ion-exclusion column (250 × 4.8 mm), maintained at 60°C using water as mobile phase with 0.8 mL/min flow rate. The HPLC system is controlled and processed by Inkarp software. Standard Formic acid and Formaldehyde solution were prepared and calibrated. Each time the product obtained is diluted with a known volume of milliq water before analysis to prevent the overloading of the column. All experiments were done in triplicates and the average values were reported within the standard deviations of <2.0%.

#### Gas chromatography-mass spectrometry (GC-MS)

The products were identified and analyzed using a GCMS-QP-2010 Ultra (M/s. Shimadzu Instruments, Japan) with a HB-5 capillary column (20 m × 0.18 mm) supplied by M/s. J&W Scientific, USA and Trace 1300 GC and ISQ qd single quadruple Mass spectrometer with a TG-5MS capillary column (30 m × 0.32 mm × 0.25 μm) supplied by Thermo Fisher Scientific, India. Gas samples were detected by Molecular sieve 5Å packed column. Thermal Conductivity Detector (TCD) was used for gas samples and Mass Detector (MSD) was used for formic acid measurement.

### NMR

^1^H NMR and ^13^C NMR spectrum is recorded on 500 MHz Bruker and 400 MHz Jeol NMR machine. For detection of formic acid by ^1^H NMR, solvent suppression (water) method is used.

#### Matrix assisted laser desorption ionization mass spectroscopy (MALDI-MS)

All samples were prepared in HPLC grade acetonitrile, by dissolving very minute amount of sample on 2 ml acetonitrile, and then filtering the sample using 0.2 micron syringe filter. Then the sample is cocrystalized with HCCA matrix.

## Result and discussion

**Figure 1:**
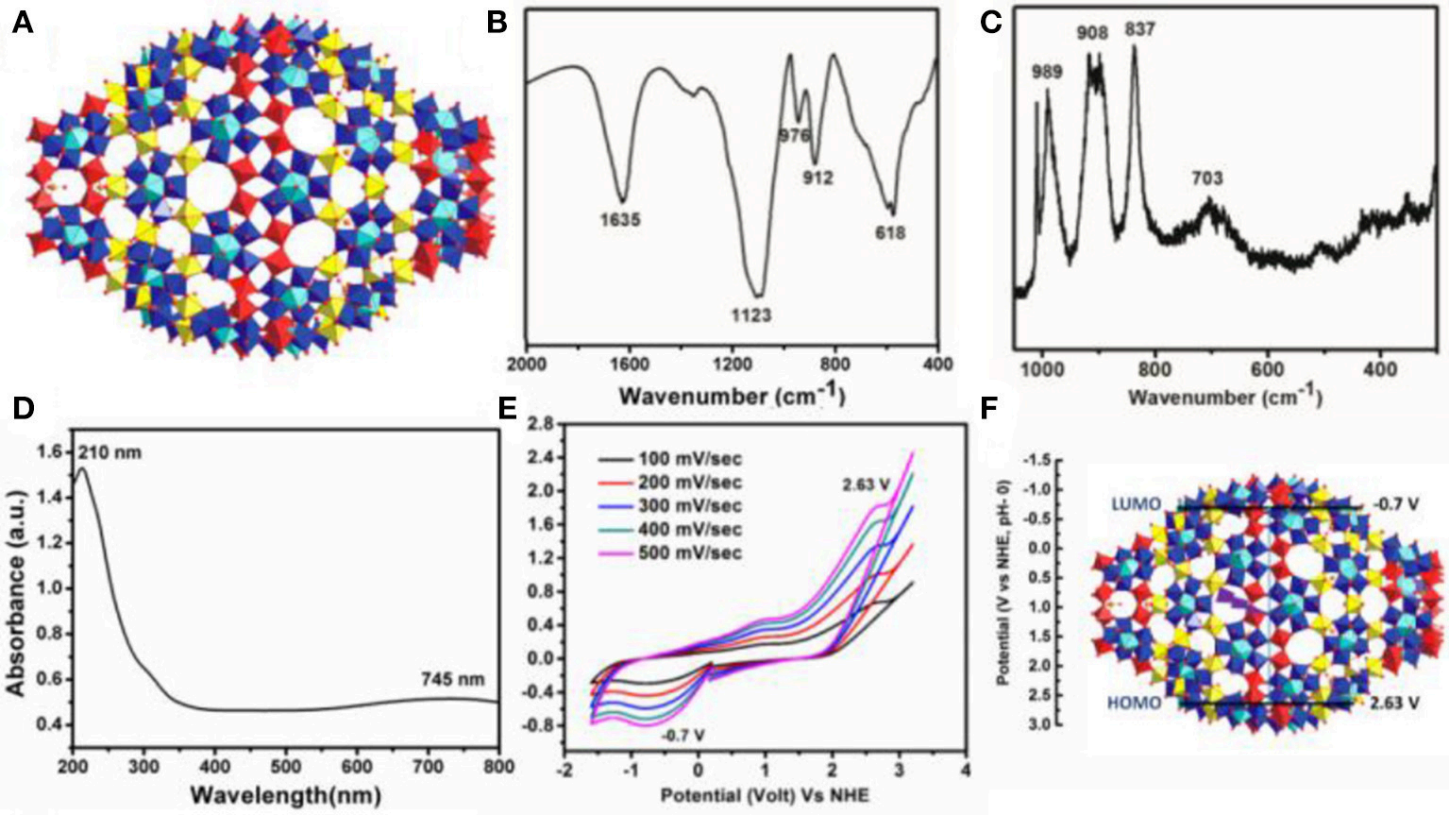
**(A)** Single crystal structure of {Mo_368_} where Red polyhedra denotes Oxygen, yellow denotes {Mo_1_} and blue with blue-turquoise pentagonal bipyramids denotes {Mo(Mo_5_)}, **(B)** FT-IR spectrum, **(C)** Raman Spectrum, **(D)** EAS spectrum, **(E)** Cyclic Voltammogram (Na_2_SO_4_ was used as electrolyte at a scan rate 100 mV/s), **(F)** HOMO and LUMO band position of {Mo_368_}.

Deep blue crystals of {Mo_368_} with *I*4mm space group are isolated from the mother liquor in a week by following the literature procedure (Müller et al., 2002). The structure was first determined by A. Müller group using single crystal X-ray diffraction. In the structure, the unit cell contains two hedgehogs like anions which possess D_4_ symmetry (Figure 1A). The complete cluster contains a central ball shaped unit {Mo_288_O_784_(H_2_O)_192_(SO_4_)_32_} and two other capping units {Mo_40_O_124_(H_2_O)_24_(SO_4_)_8_}. These two units also possess local symmetry, D_8d_, and C_4V_, respectively. The molecule has large cavity in which 400 water molecules can be encapsulated. Further this structure can be considered as a hybrid of ring shaped {Mo_176_} structure and ball shaped {Mo_102_} structure. The cluster shows typical peaks in the FTIR spectrum which are as follows (Figure 1B), 1,635 (δ_H2O_), 1,123 (ν_as_${\text{SO}}_{4}^{2-}$), 976 (νMo = Ot), 912, 618 cm^−1^, respectively. Raman spectrum (λ = 1,024 nm) shows peak at 989, 908 (broad peak), 837, and 703 cm^−1^, respectively (Figure 1C). Electronic absorption spectrum shows maximum absorbance at 747 nm which is observed due to the IVCT transition between Mo^V^ to Mo^VI^ which is characteristic for molybdenum blue species (Figure 1D). From the above all characterization details, it confirms that {Mo_368_}is formed in our reaction. Further cyclic voltammogram of {Mo_368_} under Ar atmosphere is recorded at different scan rates (Figure 1E), which gave three peaks at −0.7, 1.01, and 2.663 V, respectively vs. NHE. From the cyclic voltammogram of the material the position of HOMO and LUMO of the catalyst material was calculated. From the data, the position of HOMO is 2.63 V vs. NHE and the position of LUMO is −0.7 V vs. NHE (Figure 1F). The band gap of the catalyst material is 3.33 eV. Thus, from the band position it can be proposed that {Mo_368_} is able to reduce CO_2_ as well as oxidize water simultaneously under UV-light source of 373 nm (Figure S3). The external quantum efficiency (EQE) of the system is measured by irradiating monochromatic LED light source of wavelength 365 nm (I = 67 mW/cm^2^) and 745 nm (I = 32 mW/cm^2^) where we find the EQE of 0.6 and 1.54 × 10^−3^%, respectively (SI).

**Figure 2:**
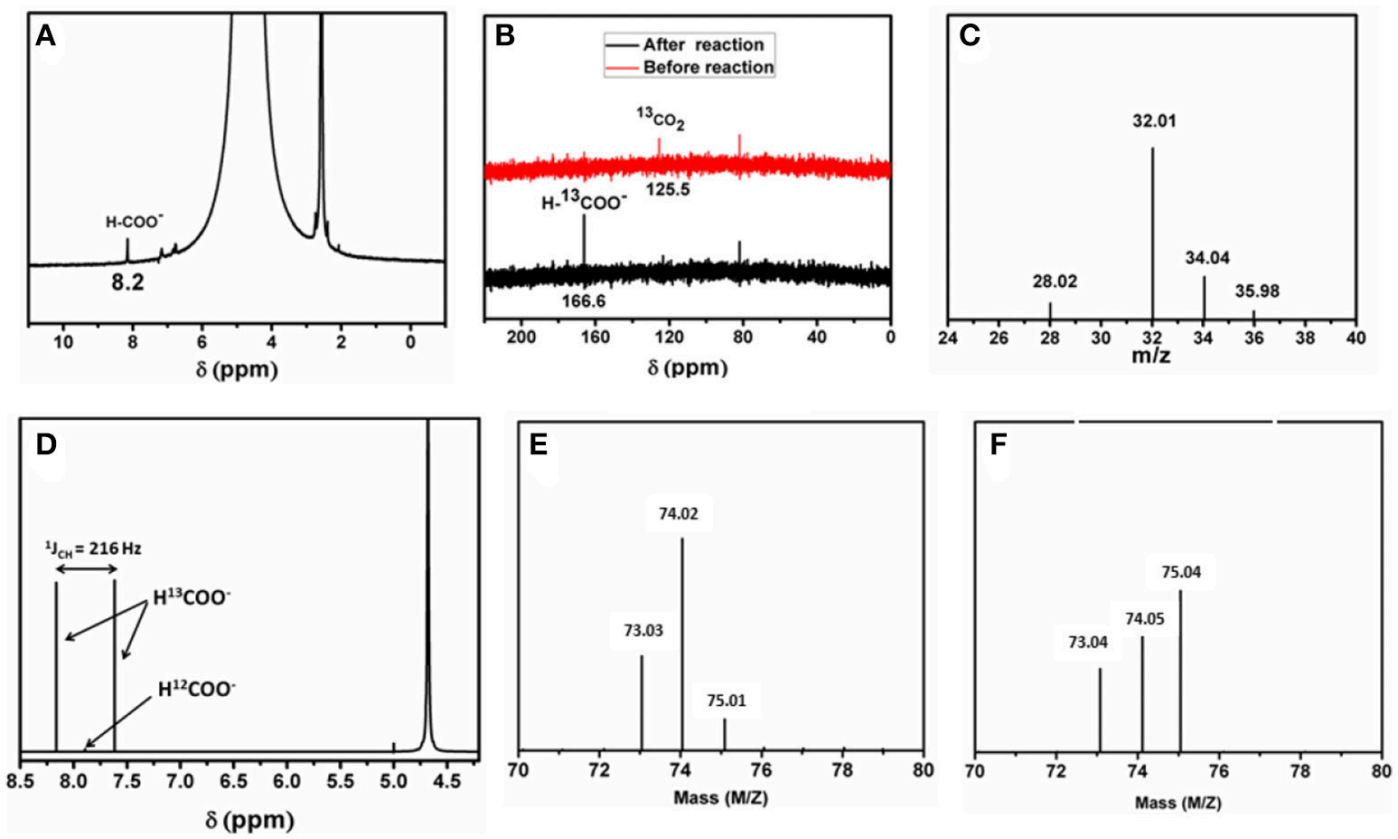
**(A)** Representative ^1^H-NMR of the product from photochemical CO_2_ reduction. **(B)** ^13^C-NMR spectrum of the reaction mixture before (red) and after (black) the reaction. **(C)** Full mass spectrum representing all the isotopes of the gaseous products (O_2_, ^16^O^18^O, ^18^O^18^O and N_2_). **(D)** ^1^H-NMR spectrum of the reaction mixture using ^13^CO_2_. **(E)** Mass of ethylformate (molecular peak) after reaction with H_2_O and **(F)** with D_2_Oand H_2_O mixture.

**Figure 3:**
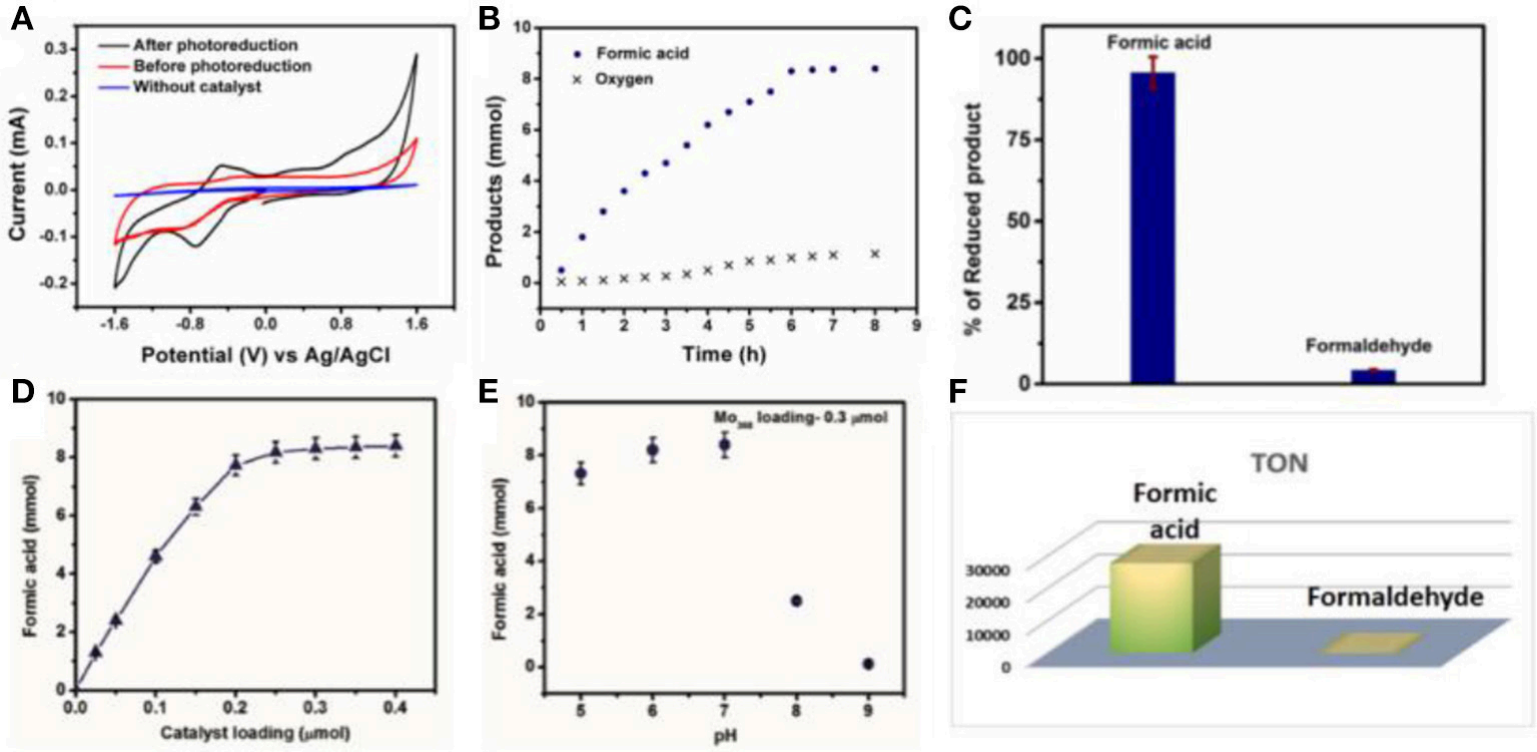
**(A)** Cyclic voltammogram of the reaction mixture, blue line corresponds to blank where there is neither CO_2_ nor catalyst, red line corresponds to CO_2_ addition without UV light illumination and black line corresponds to photo CO_2_ reduction in water using {Mo_368_} as a catalyst. **(B)** Time dependent formic acid and oxygen formation by photochemical carbon dioxide reduction using {Mo_368_} as a catalyst. **(C)** Formation of different reduced product using {Mo_368_}, and their respective percentage yield in total CO_2_ reduced product. **(D)** Effect of variation of {Mo_368_} loading on formation of formic acid. **(E)** pH dependent formic acid formation using {Mo_368_} as a catalyst. **(F)** TON of the formic acid and formaldehyde [Amount of product formed (mol)/amount of catalyst taken (mol)].

To describe the detailed catalytic process of CO_2_ reduction in water, CO_2_ reduction under UV-light without addition of any photosensitizer as well as any organic sacrificial electron donor is performed. The reaction is carried out in an air tight quartz tube which is first purged with nitrogen to remove trace amount of oxygen from the reaction system. Later CO_2_ is purged for 3 h. The quartz tube is sealed and kept in a photo reactor for different intervals of time. CO_2_ undergoes reduction under aforementioned reaction condition, the reduced product is characterized first by GC-MS and further confirmed from ^1^H-NMR by solvent suppression method (water peak suppression; Figure 2A). These above results confirm that formic acid is formed from CO_2_ in our reaction condition. Further formic acid is characterized and quantified by HPLC against a standard 0.1 M formic acid solution. A peak at −0.6 V in cyclic voltammogram is also observed using saturated Ag/AgCl as a reference electrode (Figure 3A), which further confirms the formation of formic acid in the reaction mixture. Besides formic acid, trace amount of formaldehyde which is characterized by HPLC against 0.1 M formaldehyde solution is also found. To further check whether any gaseous reduced product is formed in the reaction, GC-MS is performed by injecting the gases from the reaction chamber and no CO, CH_4_, or any other reduced gaseous product is observed from the reaction mixture. Thus, in our present reaction we get formic acid selectively over other reduced product obtained from the reduction of CO_2_. Formic acid is formed from CO_2_ only and not from any other carbon impurity. To further prove the source of carbon in formic acid, same reaction is performed using ^13^CO_2_ as a reactant yielding H^13^COOH. The product is characterized by ^13^C-NMR (Figure 2B) taken before and after reaction and Raman spectroscopy (Figure S4). Prior to UV-light irradiation ^13^C-NMR gave strong signal at 125.5 ppm corresponding to ^13^CO_2_. After the completion of 6 h of photoreduction, ^13^CO_2_ signal is found to decrease and a new peak at 166.6 ppm corresponding to H^13^COOH is obtained (Tamaki et al., 2015). We also performed ^1^H-NMR after reducing ^13^CO_2_ enriched solution where a doublet (^1^J_CH_ = 216 Hz) is found at 7.9 ppm which coupled with ^13^C atom (Figure 2D). These results indicate that formic acid is formed from CO_2_ and not from any other carbon source. This can be proved in a control experiment by recording HPLC of the light illuminated sample without purging of CO_2_. In that case, no trace of formic acid or formaldehyde is found in the reaction mixture. This indicates that formic acid and formaldehyde is obtained from CO_2_ only. During the photo catalytic CO_2_ reduction water also gets oxidized to oxygen simultaneously. Formation of oxygen is characterized by the YSI dissolved oxygen meter and GC-MS. The experiment is further repeated using mixture of ${\text{H}}_{2}^{18}$O (isotopic purity = 97%) and ${\text{H}}_{2}^{16}$O (1:8) where we found the corresponding masses at m/z = 32.01, 34.04 and 35.98. The mass *ca*. 32 corresponds to ^16^O^16^O whereas m/z = 34 and 36 resemble to ^16^O^18^O and ^18^O^18^O, respectively (Figure 2C) which confirms that oxygen is produced from oxidation of solvent water molecule. Besides, deuterium-labeling experiment was conducted using D_2_O (isotopic purity = 99.9%) and H_2_O as a solvent (3:2). We found that the deuterium was incorporated into formate moiety (m/z = 75), which was confirmed from mass spectrometry (Figures 2E,F). As exchangeable D is found in the reaction medium, it got incorporated during the formation of formic acid and later deuterium incorporated ethylformate is obtained after reaction with ethanol.

Further time dependent study reveals, 6 h is needed to complete the reduction of CO_2_ to formic acid. The TOF of the reaction is quite high 4,611 h^−1^ in water. The yield of the formic acid initially increases rapidly with time (Figure 3B). It increases almost linearly with time. After certain time of reaction, the rate of the increment of the formic acid formation with time decreases and reaction yield does not increase further after 6 h of the reaction. This indicates that the CO_2_ reduction reaction is complete within 6 h and same trend can be observed for oxygen evolution too (Figure 3B). Both the processes i.e., CO_2_ reduction and water oxidation are coupled which can be confirmed from Figure 3A where current increase occurs at 0.9 V vs. Ag/AgCl only after photoreduction. Furthermore, loading of the catalyst is varied in photochemical CO_2_ reduction in a controlled fashion (Figure 3D). Here, initially the yield of the formic acid increases almost linearly with the increasing loading of the catalyst and further after certain range of the loading of the catalyst the reaction yield becomes independent of the catalyst loading (Figure 3D). We have observed a maximum yield of 8.3 mmol of formic acid with a loading of 0.3 μmol of the catalyst with maximum turnover number of 27,666 (Figure 3F). This clearly indicates high reactivity of the {Mo_368_} unit. Other than formic acid, 37 μmol of formaldehyde is also obtained at the same loading of the catalyst under the same reaction condition. The selectivity of the formation of the formic acid with respect to the total CO_2_ reduced product is around 95.73% for formic acid and 4.27% for formaldehyde (Figure 3C). To see the effect of the proton concentration in the photo catalytic CO_2_ reduction reaction, pH is varied in the range from 5 to 9 in the course of the reaction. To do so, different buffer solutions are used (Acetate buffer was used to regulate the pH). pH dependent study reveals that the yield of formic acid is maximum at pH 7 (Figure 3E). The yield of the reaction is almost identical on moving toward acidic pH. But when we move toward the basic pH the yield of the reaction decreases drastically. This is due to the dissociation of {Mo_368_} in basic medium. Also, this is may be due to the fact that CO_2_ reduction is a proton coupled electron transfer (PCET) reaction, the reaction in basic pH, protons are quenched from the reaction medium therefore the yield of the reaction also decreases.

**Figure 4:**
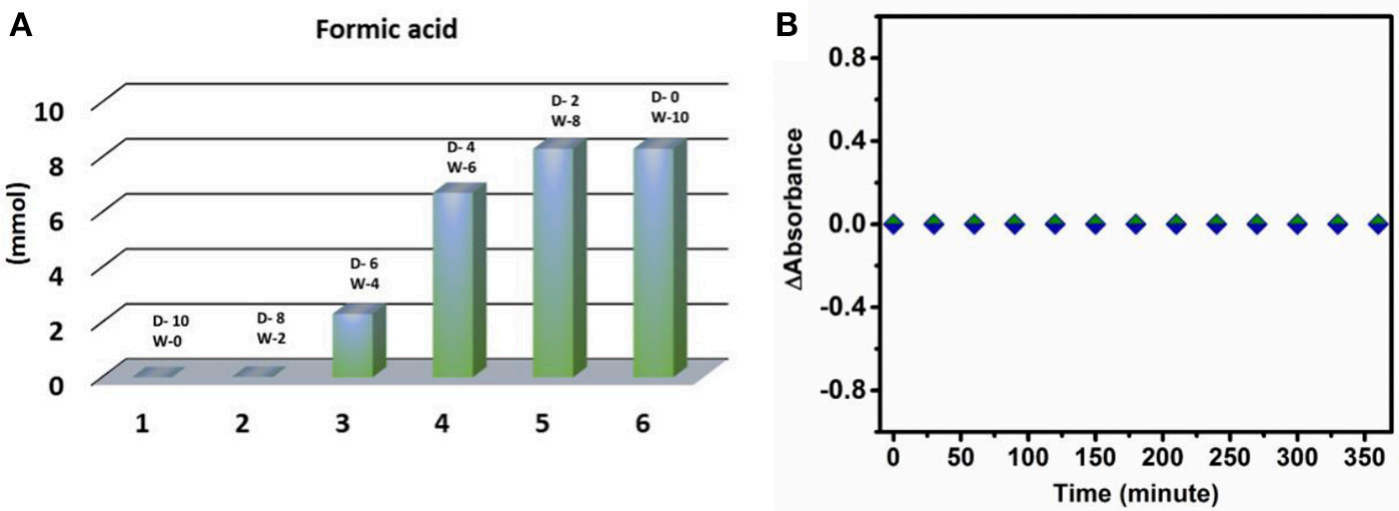
**(A)** Variation of DMF and water content in reaction mixture (D-DMF, W-water, all values given in ml unit). **(B)** Plot of change of absorbance of {Mo_368_} with time vs. reaction time.

For carbon dioxide reduction reaction two major constituents, i.e., electrons and protons are required. As no sacrificial proton donor or electron donor is used in this case, there is a possibility that water acts as a source of both protons as well as electron in the reaction. To prove this observation, different sets of experiment were performed. Initially, when the same reaction was performed in dry DMF, due to lack of availability of protons, no trace of formic acid was detected indicating that water is playing a crucial role in carbon dioxide reduction reaction under the prevalent reaction conditions (Figure 4A). Further to prove the role of the water in the photo catalytic condition, different set of reactions with varying ratio of water and DMF in the reaction mixture was carried out keeping the total volume of the reaction mixture constant (Figure 4A). From the experimental result it can be concluded that with increasing loading of water in the reaction the yield of formic acid increases linearly, which supports the role of water as proton donor.

(1) $$\begin{array}{r} CO_{2}+H_{2}O\left(aq\right)=H_{2}CO_{3} \end{array}$$

(2) $$\begin{array}{r} H_{2}CO_{3}\left(aq\right)={HCO_{3}}^{-}\left(aq\right)+H^{+}\left(aq\right) \end{array}$$

(3) $$\begin{array}{r} {CO_{3}^{2-}+4H}^{+}+2e^{-}=HCOOH\left(aq\right)+H_{2}O \end{array}$$

The dependency of water on CO_2_ reduction reaction is further obtained from pH dependent study. As, carbon dioxide reduction is proton dependent process (Equation 3), the yield of the reaction should increase on increasing the proton concentration in the reaction medium. But in our present case we observe a decrease in reduced product concentration upon going from neutral pH to acidic pH. This result indicates that another component of CO_2_ reduction reaction equilibrium i.e., electron concentration may vary with the change in the pH of the reaction. There are generally two electron sources present in reaction system: one is the catalyst material itself and another is the water. Now if the cluster were to act as electron donor in the reaction then it would degrade in the solution. If it were the situation then a decrease in the absorbance of the catalyst with increasing time of illumination may be observed. But the absorption spectrum of cluster remains unchanged throughout the reaction (Figure 4B). Moreover, in all pH variation reactions the same amount of catalyst was used resulting in a constant electronic concentration in the reaction. On the other hand, if the reaction equilibrium totally depended on proton concentration of the reaction system then the yield should have increased at lower pH. However, this does not happen in our reaction system. Hence from the above two observations it can be concluded that the catalyst material does not act as a source of electrons in the photo chemical CO_2_ reduction reaction and water must act as an electron source in the reaction.

As already discussed, oxygen formation was detected in the system during CO_2_ reduction which proves that the oxidation of water releases electrons into the system. As it is known that the water oxidation reaction depends on the pH of the medium and it increases with increasing pH of the reaction i.e., ongoing from the acidic to basic pH water oxidation increases. As water oxidation increases at higher pH electron concentration also increases which will facilitate the reduction process. Thus, CO_2_ reduction should increase at higher pH. It is also observed in our present reaction system that CO_2_ reduction increases with increasing pH (Up to pH 7, as at basic pH the catalyst dissociates). Thus, from the above two observations we can conclude that water only acts as a sacrificial electron donor in the reaction and photo chemical CO_2_ reduction depends on photo-chemical water oxidation reaction which also takes place parallelly in the reaction system. Thus, a maximum yield of formic acid is obtained in neutral pH as compared to acidic pH.

To investigate the active catalyst species in the reaction, the reaction is repeated with the precursor of {Mo_368_} i.e., with sodium molybdate and no reduced product is found in the reaction. Thus, from this result, it can be concluded that the giant cluster {Mo_368_} is only responsible for CO_2_ reduction reaction and not a single molybdenum unit. Cluster cage thus plays a crucial role for the reaction. It is already mentioned that {Mo_368_} is a photoactive materialand it absorbs UV-light of 373 nm wave length to generate holes (h^+^) and electrons (e^−^) in the system. These holes can oxidize water to generate electrons, protons and oxygen in the medium. The electrons and protons so generated reduce CO_2_ to formic acid and formaldehyde. This also explains the high photo catalytic activity of {Mo_368_}. {Mo_368_} comprises of different small molybdenum based units i.e., {Mo^VI^(${\text{Mo}}_{5}^{\text{VI}}$)}, {${\text{Mo}}_{2}^{\text{V}}$}, {${\text{Mo}}_{1}^{\text{V}}$}, respectively. We believe that upon photo excitation, {Mo^VI^(${\text{Mo}}_{5}^{\text{VI}}$)} goes to excited state and forms {Mo^VI^(${\text{Mo}}_{5}^{\text{VI}}$)}^*^. This species has potential to oxidize water to liberate oxygen and release protons and electrons. On the other hand, the {Mo_2_} unit and {Mo_1_} unit plays a crucial role for photochemical CO_2_ reduction. It has been already shown by Müller group that CO_2_ can coordinate with {Mo_2_} units of the giant molybdenum based polyoxometalates (Garai et al., 2012; Bandeira et al., 2015). Thus, in our present case it is also reasonable to postulate that CO_2_ can co-ordinate with {Mo_2_} unit. It is also observed that there is change in cyclic voltammogram of {Mo_368_} upon purging of CO_2_ in {Mo_368_} solution (Figure S2). Further this coordinated CO_2_ unit can be reduced by electrons and protons present in the reaction medium. Possibly CO_2_ also can coordinate with {Mo_1_} unit and reduction of coordinated CO_2_ takes place in a synergistic fashion. We believe that adsorbed CO_2_ converted to C$O_{2}^{-\cdot }$. The activation barrier of this reduction is lowered by the cluster. Later this species accept proton to generate carboxyhydroxyl intermediate species which participates in PCET process to form formic acid. Due to direct attachment, a large number of CO_2_ molecules are reduced for every molecule of {Mo_368_}, and hence the cluster shows such high photocatalytic activity.

**Figure 5:**
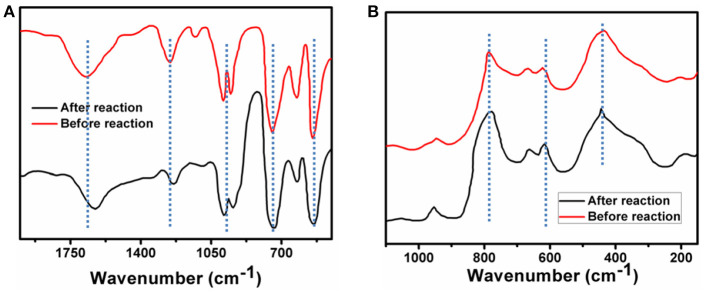
**(A)** FT-IR spectrum of {Mo_368_}, black line corresponds to FT-IR spectrum of before reaction and brown line corresponds to FT-IR spectrum of after reaction. **(B)** Raman spectrum of {Mo_368_}, black line corresponds to Raman spectrum of before reaction and brown line corresponds to Raman spectrum of after reaction.

The stability of the catalyst is discussed under prevalent photo catalytic conditions. After reaction the catalyst is recovered by slow evaporation of solvent. In this present case it is shown that the {Mo_368_} is stable under the photocatalytic condition using different techniques. FT-IR spectrum of {Mo_368_} after completion of the reaction shows peaks at 1632, 1124, 985, 916, 615 cm^−1^, respectively which are almost similar to that of the solid catalyst. This indicates that the cluster is stable under the reaction conditions (Figure 5A). Moreover, the IVCT bands responsible for the Mo(V)->Mo(VI) transitions do not change with the reaction and it further indicates that there is no change in the catalyst composition and that the catalyst is stable under the reaction conditions. Similar result is also observed from Raman spectrum of catalyst which also shows the catalyst remains intact after the photochemical CO_2_ reduction (Figure 5B). Also note that no presence of particulate matter is detected from DLS in this system during catalysis which indicates that the catalyst is homogeneous. Hence our catalyst is a stable homogeneous catalyst which reduces CO_2_ to formic acid in water.

## Conclusion

Photochemical reduction of CO_2_ to formic acid in water using molybdenum based giant polyoxometalate {Mo_368_} molecular catalyst is reported. The photocatalytic system shows excellent selectivity for formic acid production (95.73%) with high TON (27,666) and TOF (4,419 h^−1^); which is quite high in its class. The presence of different Mo based sub units in the cluster is responsible for the exceptional activity of the catalyst. As the system is photoactive, no external photosensitizer is added and water solvent acts as electron donor making the whole process environmentally benign and self-sustained. We believe that this work can lead to the development of a class of highly efficient homogeneous CO_2_ reduction catalysts based on water soluble polyoxometalates.

## Author contributions

SD, TB and SB contributed equally. SD, TB and SB performed the experiments. SS assisted in those experiments. RP carried out GC-MS and HPLC experiments. SR designed the project, analyzed the results and wrote the paper with inputs from all other co-authors.

### Conflict of interest statement

The authors declare that the research was conducted in the absence of any commercial or financial relationships that could be construed as a potential conflict of interest.
