# Corrigendum: A Molecular CO_2_ Reduction Catalyst Based on Giant Polyoxometalate {Mo_368_}

**DOI:** 10.3389/fchem.2021.666341

**Published:** 2021-06-24

**Authors:** Santu Das, Tuniki Balaraju, Soumitra Barman, S. S. Sreejith, Ramudu Pochamoni, Soumyajit Roy

**Affiliations:** ^1^ Eco-Friendly Applied Materials Laboratory, College of Chemistry, Central China Normal University, Wuhan, China; ^2^ Eco-Friendly Applied Materials Laboratory, Department of Chemical Sciences, Materials Science Centre, Mohanpur, Indian Institute of Science Education and Research, Kolkata, India

**Keywords:** CO_2_ reduction, polyoxometalate, homogeneous catalysis, water oxidation, photochemistry

In the original supplementary material of the article, there was an error in **Figure S1** as published. MALDI mass spectrum is replaced with GC-MS spectrum. The ^1^H NMR spectra is replaced with the ^1^H NMR spectra of the isolated product. The updated **Figure S1** and its caption can be seen below.

## Supplementary Material

The authors apologize for this error and state that this does not change the scientific conclusions of the article in any way. The original article has been updated.

