# Supplementary material for: A Molecular CO2 Reduction Catalyst Based on Giant Polyoxometalate {Mo368}
Source: Front Chem. 2018 Nov 2;6:514. doi: 10.3389/fchem.2018.00514 (PMC6224680; doi:10.3389/fchem.2018.00514)
Supplement: Supplementary file 1 [file Data_Sheet_1.PDF]

Supporting online material

A Molecular CO_2 Reduction Catalyst Based on Giant Polyoxometalate $\{Mo_{368}\}$

*Santu Das, Tuniki Balaraju, Soumitra Barman, S.S. Sreejith, Ramudu Pochamoni, Soumyajit Roy**

EFAML, College of Chemistry, Central China Normal University,152 Luoyu Road, Wuhan, 430079 Hubei, P. R. China.

And

Eco-Friendly Applied Materials Laboratory (EFAML), Materials Science Centre, Department of Chemical. Sciences, Mohanpur Campus, Indian Institute of Science Education & Research, Kolkata, 741246 West Bengal. s.roy@iiserkol.ac.in, roy.soumyajit@gmail.com

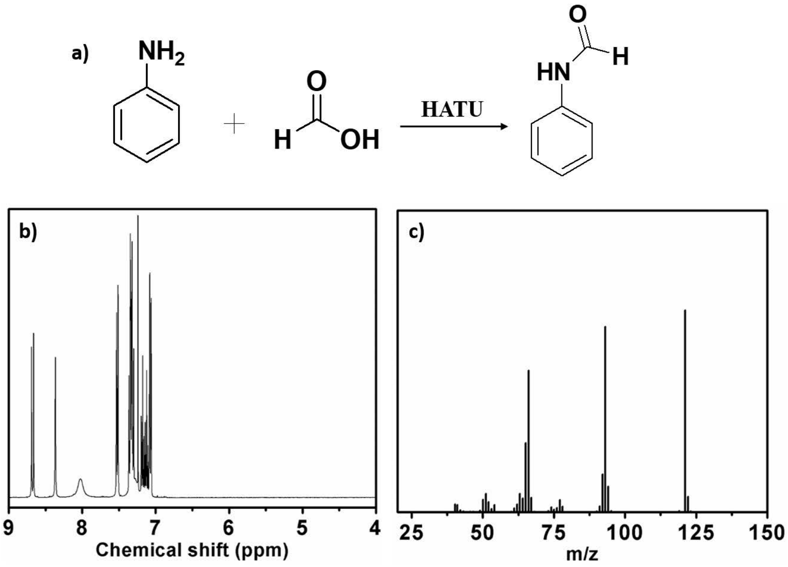

Fig. S1. a) Reaction of aniline with formic acid using HATU (1- [bis(dimethylamino) methylene]-1H-1,2,3-triazolo [4,5-b] pyridinium 3-oxid hexafluorophosphate) as coupling agent b) 1H NMR spectrum of purified N-phenylformamide c) Mass spectrum of N-phenylformamide obtained from GC-MS.

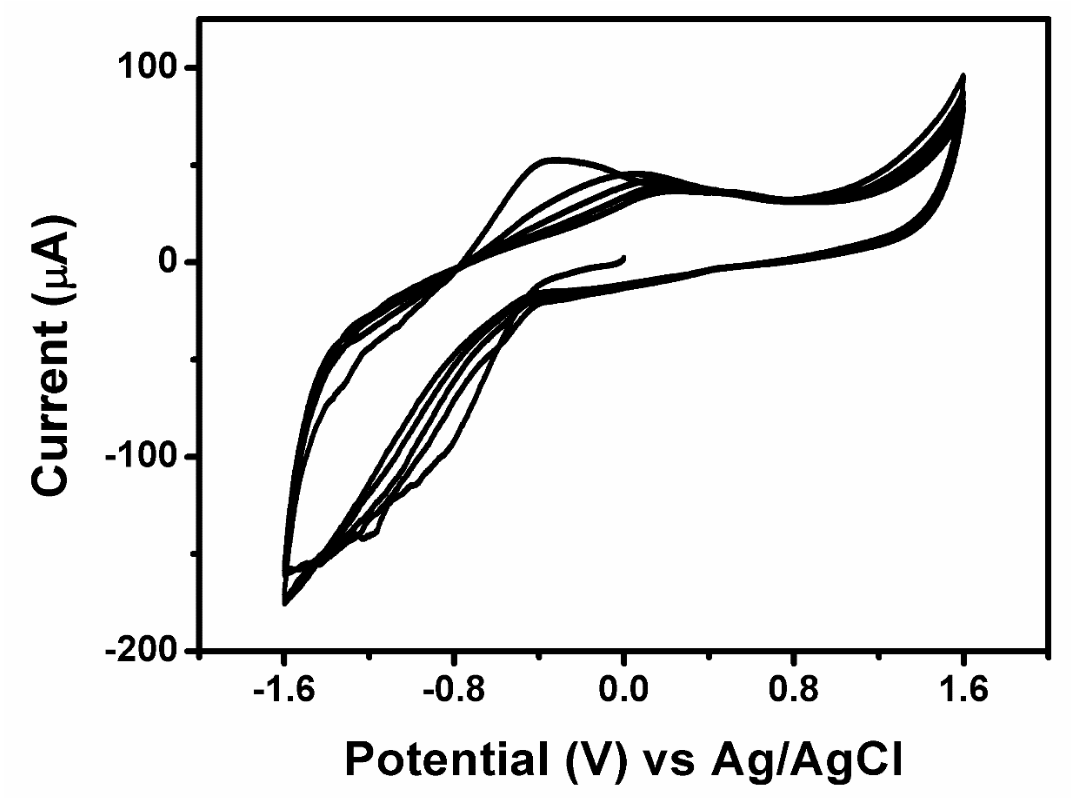

Fig. S2. Cyclic Voltammogram on the effect of CO_2 on the catalyst at the scan rate 100 mV/s. (0.1 M Na_2SO_4 is used as the electrolyte)

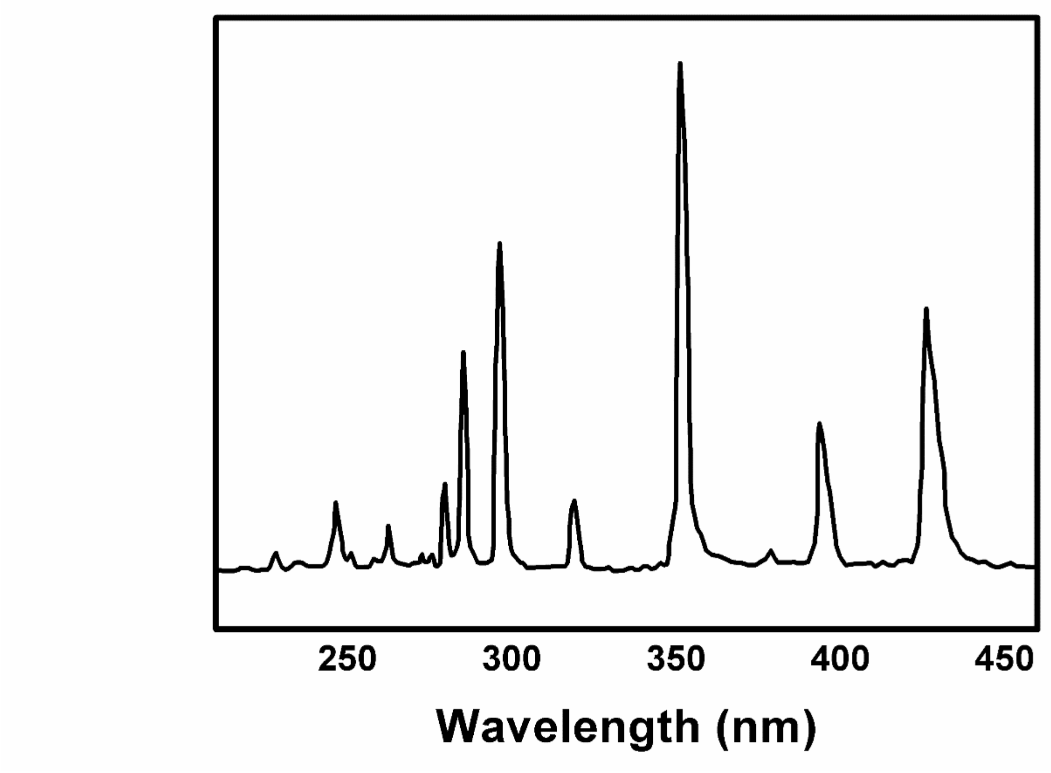

Fig. S3. Representative spectrum of the UV lamp used for the photochemical reaction.

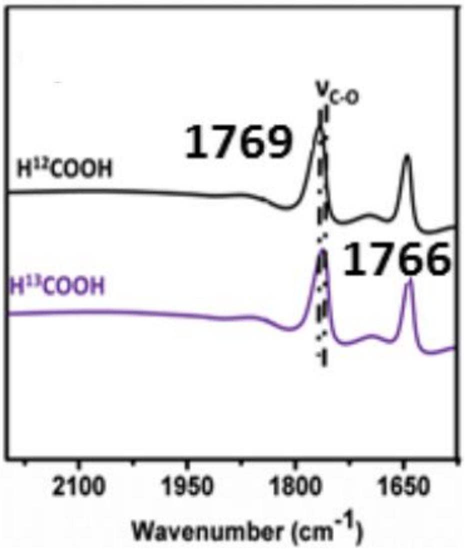

Fig. S4. Raman spectrum of $H^{12}COOH$ (Black line) and $H^{13}COOH$ (violet line) from post reaction solution[1].

Calculation of external quantum efficiency (EQE):

Photocatalytic CO_2 reduction reaction was carried out by illuminating two different monochromatic light of wavelength 365nm (UV, 67 W/cm^{-2}) and 745nm (Visible, I=32mW/cm^{-2}) at 25°C. We calculated the EQE using the following formula[2, 3]

$$EQE\ (\%) = (2.n_{HCOOH}.N_{A}.h.c)/(t_{irr}.\lambda.I.A)$$

Where n_{HCOOH} is the moles of formic acid produced, N_A is the Avogardo constant, h is the Planck constant, c is the speed of light, t_{irr} is the irradiation time, A is the irradiated area of the photoreactor.

References

1. R. Bartholomew and D. Irish, *Journal of Raman spectroscopy*, 1999, **30**, 325-334.
2. C. A. Caputo, M. A. Gross, V. W. Lau, C. Cavazza, B. V. Lotsch and E. Reisner, *Angew. Chem. Int. Ed.*, 2014, **53**, 11538-11542.
3. J. J. Walsh, C. Jiang, J. Tang and A. J. Cowan, *PCCP*, 2016, **18**, 24825-24829.
